# Carbon Nanomaterials Based Smart Fabrics with Selectable Characteristics for In-Line Monitoring of High-Performance Composites

**DOI:** 10.3390/ma11091677

**Published:** 2018-09-11

**Authors:** Guantao Wang, Yong Wang, Yun Luo, Sida Luo

**Affiliations:** 1 Department of Material Processing and Controlling, School of Mechanical Engineering & Automation, Beihang University, Beijing 100191, China; wangtgt@cugb.edu.cn (G.W.); yongw@buaa.edu.cn (Y.W.); 2 School of Engineering and Technology, China University of Geosciences (Beijing), Beijing 100083, China; luoyun@cugb.edu.cn

**Keywords:** carbon nanomaterials, smart fabrics, in-line monitoring, polymeric composites, carbon nanotubes, reduced graphene oxide

## Abstract

Carbon nanomaterials have gradually demonstrated their superiority for in-line process monitoring of high-performance composites. To explore the advantages of structures, properties, as well as sensing mechanisms, three types of carbon nanomaterials-based fiber sensors, namely, carbon nanotube-coated fibers, reduced graphene oxide-coated fibers, and carbon fibers, were produced and used as key sensing elements embedded in fabrics for monitoring the manufacturing process of fiber-reinforced polymeric composites. Detailed microstructural characterizations were performed through SEM and Raman analyses. The resistance change of the smart fabric was monitored in the real-time process of composite manufacturing. By systematically analyzing the piezoresistive performance, a three-stage sensing behavior has been achieved for registering resin infiltration, gelation, cross-linking, and post-curing. In the first stage, the incorporation of resin expands the packing structure of various sensing media and introduces different levels of increases in the resistance. In the second stage, the concomitant resin shrinkage dominates the resistance attenuation after reaching the maximum level. In the last stage, the diminished shrinkage effect competes with the disruption of the conducting network, resulting in continuous rising or depressing of the resistance.

## 1. Introduction

By virtue of remarkable features, including high specific modulus and strength, adiabaticity, corrosion resistance, and shock absorption [1], fiber-reinforced polymeric composites (FRPs) have been applied in widespread fields, such as aerospace technology, automotive industry, shipbuilding, and civil engineering. The booming applications of FRPs have inevitably caused people to worry about their quality and life assurance [2]. Under such circumstances, structural health monitoring (SHM) has emerged as a scientific and necessary tool, playing an important role in identifying, quantifying, and deciding the health states of the high-performance composites. In addition to traditional methods, such as strain gages [3], optical fibers [4], metal oxide films [5], guided waves [6,7,8], and piezoelectric sensors [9], carbon nanomaterials are considered to be novel materials for establishing embeddable, built-in, lightweight, versatile, and flexible self-sensing technology for composites [10]. Because of their extraordinary mechanical robustness, structural non-invasiveness, and piezoresistive sensitivity [11], it is important to investigate the great potentiality of novel carbon nanomaterials-based sensors to improve the structural health monitoring performance of composites. In addition, the key carbon nanomaterials, such as carbon nanotubes (CNTs) and graphene, are being adopted for multiple SHM purposes for FRPs. For examples, CNTs and graphene powders have been respectively mixed in resin matrix to monitor the failures and damages of the FRPs under mechanical deformations [12,13]; CNT/graphene-based films [14,15,16] and papers [17] have been placed on top of or embedded between lamination layers as strain sensors for deformation detection. Graphene-coated fibers have also been utilized for monitoring tensions and/or compressions [18].

Although most of the current research on carbon nanomaterials-based sensing technology mainly focuses on SHM during the service stages of the composites, it is equally important to monitor the manufacturing stage to detect and guide resin infiltration and curing, which are significant for assuring the quality and performance of the final products [19]. Considering the off-line limitations of differential scanning calorimetry (DSC), rheological, spectroscopic [20], and simulation methods [21], carbon nanomaterials could also serve as novel in-line monitoring strategies to meet the standards and requirements of composite manufacturing. In this respect, Lu et al. [2,19] utilized CNT buckypapers to monitor the real-time cure behavior of FRPs; for reducing part-to-part variations, Gnidakouong et al. [13,22] monitored and assessed the resin flow and curing levels of CNT/fiber-enabled polyester composites in a vacuum-assisted resin transfer molding (VARTM) process. Ali et al. [23] exploited graphene-coated piezo-resistive fabrics for monitoring the process of liquid composite molding. Our group recently enabled CNT and graphene thin films coated on reinforcement fibers to monitor and quantitatively analyze the composite manufacturing under both dry [24,25] and liquid [26] molding processes.

Summarizing the sensing performance of all the carbon nanomaterials-based sensors, it is surprising that the maximum resistance change has varied substantially from ~30% for graphene-based sensors [23] to ~1600% for CNT-based sensors [26]. To investigate the possible structure–property relationship, this paper focuses on the systematic analysis and comparison of three types of carbon nanomaterials-based sensors for monitoring the VARTM process of FRPs, namely, CNT-enabled fabrics (CNTF), reduced graphene oxide (RGO)-enabled fabrics (RGOF), and carbon fiber (CF)-enabled fabrics (CFF). Specifically, a high-efficient fiber winding and coating system was established for coating CNTs or graphene on fiber substrates. Through scanning electron microscopy (SEM) and Raman analysis, the microstructures of various carbon nanomaterials-based fibers were characterized in detail. For instance, a CNT-coated fiber has a loose and disordered packing structure with visible pores. In comparison, RGO coating is ultrathin and conformal with large lateral dimensions. CF itself is composed of continuous and densely packed graphites. The as-produced fiber sensors were respectively braided into a fiber fabric as the smart fabric layer of the composites. Through complete monitoring of the resin infiltration and curing, the piezoresistive behavior of the smart fabrics was divided into three stages (i.e., the gelation stage, hardening stage, and post-curing stage). Significant dissimilarities in sensing performance can be found at each stage after comprehensive analysis of various fabrics, which leads us to be more convinced of the existence of selectable structure-dependent characteristics driving the generation of various sensing responses. Briefly, in the gelation stage, the packing structure of the embedded fiber sensors dominates the increasing level of resistance through the resin infiltration; in the hardening stage, the resistance attenuation is closely linked to the resin shrinkage, causing the densified conducting network; and in the post-curing stage, the diminished shrinkage effect competes with the defects of the packing structure, resulting in the continuous rising or depressing of the resistance.

## 2. Materials and Methods

### 2.1. Materials

In this work, multi-walled carbon nanotubes (MWCNTs, General Nano LLC., Cincinnati, OH, USA) were used as coating materials for the fabrication of CNTF. Graphene oxide (GO) was synthesized via the Hummers’s method and used for assembling RGOF [27]. Triton^TM^ X-100 (CAS # 9002-93-1, Sigma-Aldrich, Beijing, China) was selected as surfactant for dispersing CNTs in the aqueous solution. A hydroiodic acid solution (CAS # 10034-85-2, Sigma-Aldrich, Beijing, China) was utilized for the GO reduction. Fiber bundles with lengths of 15 cm, drawn from plain-woven glass fabrics (Part # GF-PL-290-100, Easy Composites Ltd., Beijing, China), were used as substrates for fiber coatings. CF rovings (Part # CF-PL-210-100) with lengths of 15 cm were also acquired from the Easy Composites Ltd.(Beijing, China). The above-mentioned glass fabrics were also used for reinforcing composites.

### 2.2. Preparation of Smart Fabrics 

**Figure 1:**
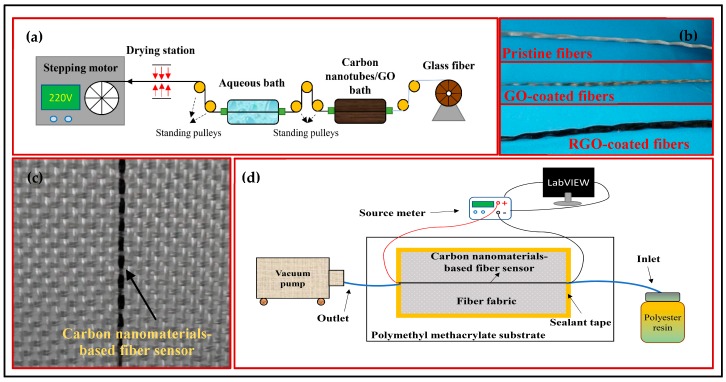
(**a**) Schematic diagram of the fiber winding and coating system. (**b**) Photographs of the pristine, graphene oxide (GO) and reduced graphene oxide (RGO)-coated fibers. (**c**) A representative smart fabric with an embedded carbon nanomaterials-based fiber sensor. (**d**) The schematic diagram of the setup for the monitoring of the vacuum-assisted resin transfer molding (VARTM) process.

Following established strategies of materials’ exfoliation [28,29], MWCNTs (300 mg) were sonicated in deionized water (100 mL) with 5 mL of Triton X-100 surfactant for 120 min, using a Ultrasonics FS-600N probe sonicator operated in a pulse mode (on 10 s, off 10 s), with the power fixed at 480 W. Following the same conditions as in the sonication process, the GO dispersion was prepared with 300 mg of GO powders in 100 mL of deionized water. Based on our previous works [10,26], a modified fiber winding and coating system was established, as shown in Figure 1a, in which the fiber powertrain was made up of a stepping motor and multiple standing pulleys for CNT/GO bathing, aqueous cleaning, and thermal drying. After the coating process, the GO-coated fibers required an additional reduction procedure to form the RGO-coated fibers by dipping the fibers into a hydroiodic acid solution at 85 °C for 30 min. With a clear contrast of colors, Figure 1b visually compares the appearance of the RGO-coated fibers at three fabrication stages, including pre-coating (white), post-coating (dark yellow), and post-reduction (black). Then, to obtain the smart fabrics, the as-produced fiber sensor was embedded in a plain-woven fabric (15 cm × 15 cm) by manual extracting and weaving, as shown in Figure 1c.

### 2.3. In-Line Process Monitoring of Composites

A VARTM process was performed with the embedded smart fabrics for the in-line monitoring. Figure 1d schematically shows the experimental setup. Together with 2 layers of pristine fabrics, the smart fabric layer was first stacked on a polymethyl methacrylate substrate. To facilitate the electrical measurements, copper tapes were used as electrodes connecting both ends of the fiber sensor with a silver paste. Then, a peel ply (ELS60100, Airtech Ltd., Tianjin, China) and a flow mesh (ELS60100, Airtech Ltd., Tianjin, China) were laid sequentially on the fabric layers for guiding the resin fluid flowing through the two nylon tubes fixed as the inlet and outlet. Assisted by a double-sided sealant tape (AT200Y1/250, Airtech Ltd., Tianjin, China), a vacuum bagging film (WL5400, Airtech Ltd., Tianjin, China) was used for sealing all the preforms.

After setting up the preforms, a vacuum pump was continuously running to introduce the polyester resin (IP2, Easy Composites Ltd., Beijing, China) mixed with 1.5 wt.% hardener (methyl ethyl ketone peroxide, MEKP, Easy Composites Ltd., Beijing, China) to infuse, infiltrate, and finally, fill the above-created vacuum space. Based on the guidance of the resin product, the manufacturing process lasted 24 h. During this period, the resistance of the embedded fiber sensor was recorded in real time by a source meter (2450, Keithley Ltd., Shanghai, China) interfaced with a homemade LabVIEW program. For each type of fiber sensor, at least 5 samples assembled by the same recipe and condition were tested to study reproducibility of the performance.

### 2.4. Structural Characterization and Performance Evaluation

To characterize the microstructure of the fiber sensor, scanning electron microscopy (SEM) and Raman spectroscopy were employed. The SEM was operated by a JEOL-JSM-7001F at 20 kV. The light source of the Raman microscope (Horiba-HR800, Horiba Ltd., Kyoto, Japan) is a 532-nm excitation laser controlled at 5 mW. In this work, the relative resistance change (*dR/R0*) was utilized to describe the sensitivity of the various smart fabrics [19], where *R0* is the initial resistance. The initial resistance of all the CNT- and RGO-coated fibers and the CFs with lengths of 15 cm in the experiments were respectively stabilized at ~200 kΩ, ~400 kΩ, and ~20 Ω.

## 3. Results and Discussion

### 3.1. Microstructure of Carbon Nanomaterials-Coated Fibers 

**Figure 2:**
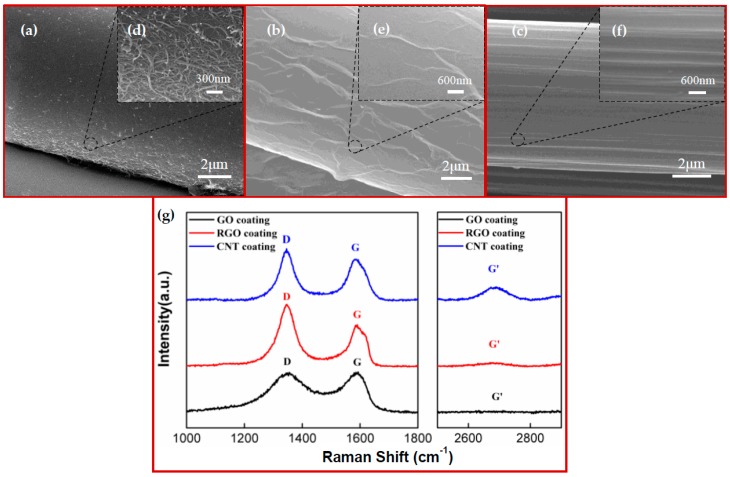
(**a**–**c**) SEM images of the CNT- and RGO-coated fibers, as well as CF, at a low magnification. (**d**–**f**) SEM images of CNT- and RGO-coated fibers, as well as CF, at a high magnification. (**g**) Raman spectrum of GO, RGO, and CNT coatings.

A detailed structural characterization of the embedded carbon nanomaterial-coated fibers is essential for exploring the structure-dependent characteristics of the smart fabrics. SEM was therefore requisitioned to examine the morphology and microstructure of the various sensors. Figure 2a–f show the SEM micrographs of the CNT- and RGO-coated fibers from multiple perspectives, as well as CF. The images at low magnification displayed the overall packing structures and were used for viewing the coating effectiveness. To specify, with respect to the CNT-coated fibers, large numbers of disordered CNT particles were intertwined and agglomerated together, forming a uniformly distributed coating layer on the filament substrate (Figure 2a). For the RGO-coated fibers, flexible graphene flakes were stacked in a staggered manner to splice a soft sheet tightly wrapping around the fiber (Figure 2b). As for CF, its highly consistent and continuous graphite structure was clearly visualized in Figure 2c [30]. More detailed structural information can be found in Figure 2d–f under high magnifications. With one-dimensional tubular structures, the fluffy CNTs were randomly entangled, introducing abundant porous structures throughout the network (Figure 2d). In contrast, the RGO possessed two-dimensional plate-like structures, making it easy to spread out on fiber surfaces [31]. Caused by layer-by-layer assembly, the wrinkles and small gaps between the neighboring flakes can be observed in Figure 2e. In Figure 2f, the graphite crystallites were seamlessly packed along the axial direction of the fiber and exhibited clear ridges.

To further study the structural properties, Raman spectroscopy was explored to characterize the crystal structures of the carbon nanomaterials [32,33]. Figure 2g shows the Raman spectra of the CNT, RGO, and GO coatings. It was observed that all the samples presented similar Raman features with three characteristic peaks, confirming the existence of CNT and graphitic structures. Namely, the G peak appeared in the range of 1585–1595 cm^−1^ and was caused by the stretching of the C–C bonds in the graphitic structures corresponding to the first-order scattering of the E_2g_ mode [34,35]. The D peak, located around 1350 cm^−1^, was a defect-induced Raman feature [36], which was closely related to the defects and crystal distortion [37]. The G’ peak, located around 2700 cm^−1^, originated from a double resonance Raman process [38], providing related information on the energy band structure of the carbon-based materials. As clearly exhibited in Figure 2g, the widths of the D and G peaks in the spectrum of the GO were broader than that of the RGO and CNTs, possibly due to the introduction of the disordered sp^3^ carbon by breaking the symmetrical and ordered sp^2^ structures in the graphite during the extensive oxidation. From the GO to the RGO, a clear enhancement in the intensity of the D peak can be observed. As stated above, this suggests the increased number of defects on the graphene flakes by the massive removal of the oxygen-containing groups through reduction. Accordingly, we have observed the increase in the intensity ratio between the D and G peaks (I_D_/I_G_). In accordance with other studies [35,39,40], this may be caused by the decrease in the average crystallite size of the sp^2^ carbon domains in the RGO, because large numbers of sp^3^ carbons are deoxygenated to form new sp^2^ domains. Additionally, the intensity of the G’ peak in the spectrum of the RGO is much weaker than that of the CNTs, indicating the formation of considerable defects in the double resonance process and disclosing the multi-layered structure of the RGO [37,41,42].

### 3.2. Piezoresistivity of Fiber Sensors for In-Line Monitoring 

**Figure 3:**
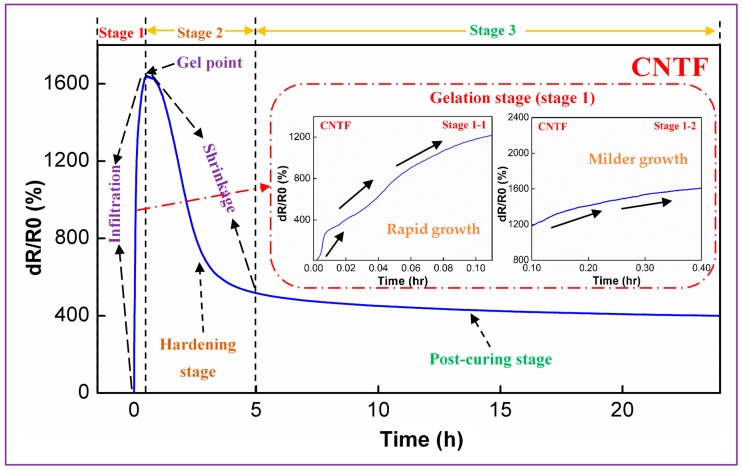
Representative sensing performance of a CNT-enabled fabric (CNTF) for the in-line monitoring of fiber-reinforced polymeric composites (FRP) manufacturing.

The conducting network created by the carbon nanoparticles attached to the fiber surface endows the fabrics with sensorial capabilities [18]. To explore the process monitoring characteristics and understand the structure-dependent mechanisms, each of the fabric sensors was applied to an in situ monitor to track the complete VARTM by recording the electrical resistance in real time. To demonstrate this idea, the real-time resistance change (*dR/R0*) of a representative CNT-enabled fabric (CNTF) is shown in Figure 3. To better understand the sensing behavior, we have divided the signal curve into three parts according to the key stages of the resin curing process.

As shown in Figure 3, *dR/R0* keeps changing throughout the whole process of composite molding. In the first 0.4 h, a significant increase of *dR/R0* can be clearly observed, correlating closely with the resin infiltration by rapidly entering the dry reinforcement to gradually fill the entire space under the vacuumed condition. Interestingly, this time interval matches exactly the gel time provided by the resin supplier (~25 min) and thus is named the gelation stage (stage 1). In addition, the piezoresistivity of this stage can be further subdivided into two small stages, namely, stage 1-1 (0–0.1 h) with rapid resistance growth and stage 1-2 (0.1–0.4 h) with a milder increase of *dR/R0*. As the molding process proceeds, the manufacturing transitions into the hardening stage (stage 2) due to the occurrence of a drastic crosslinking reaction. In this stage, *dR/R0* begins to decrease substantially until the time reaches about 5 h, indicating the gradually weakened reaction of crosslinking. Subsequently, the final stage starts, which lasts until the end of the process (5–24 h). It is called the post-curing stage (stage 3), because the degree of curing does not change drastically and *dR/R0* also gradually approaches a stabilized value. 

The abovementioned piezoresistive effect may be strongly associated with the phase change of polyester resin during the curing process. As investigated in the research of Chiacchiarelli et al. [43], the piezoresistivity of the carbon nanomaterials is dominated mainly by the numbers of conductive pathways and the variation of contact resistance between adjacent particles. In the gelation stage, with the elapse of time, the resin viscosity gradually changes from low to high and eventually loses its fluidity. In this context, the flow rate of the resin determines the increasing rate of *dR/R0*. At the beginning of the infusion, the lowest viscosity along with almost unobstructed space makes the resin flow swiftly to fill the vacuumed space. This brings about a rapid rise in *dR/R0* (0–~1200%) by expanding the distance between adjacent CNTs to deteriorate and even completely damage the interconnection of the conductive particles (stage 1-1). After filling the majority of the voids, the resin could infiltrate to the internal space of the fiber bundles to further improve the fiber wetting. At this point, the increased viscosity, as well as the reduced space, simultaneously slows down the resin flow [13]. Thus, the effect of the electrical disturbance is significantly reduced, leading to a gentle rise in *dR/R0* (~1200%–~1600%, stage 1-2). By meeting the gelation time, the resin flow almost stops, making the value of *dR/R0* stay temporarily in a stabilized state. In the hardening stage, the resin evolves from the gel state to the deep crosslinking state, causing certain levels of volume shrinkage [44]. Suffering from the matrix shrinkage, as well as internal stress, the impregnated nanoparticles become closer, re-connected, or even tightened. As a result, the attenuation of *dR/R0* (from ~1600% to ~517%, stage 2) has been observed. Once the resin matrix can maintain its shape and hardness, it will enter the post-curing stage, in which the resin slowly evolves to a finalized product with optimized structural properties. Resultingly, the structure of the electrical network is merely disrupted, and its *dR/R0* decays slowly and stabilizes eventually (between ~517% and ~401%, stage 3).

### 3.3. Comparison of Sensing Performance of Various Smart Fabrics

**Figure 4:**
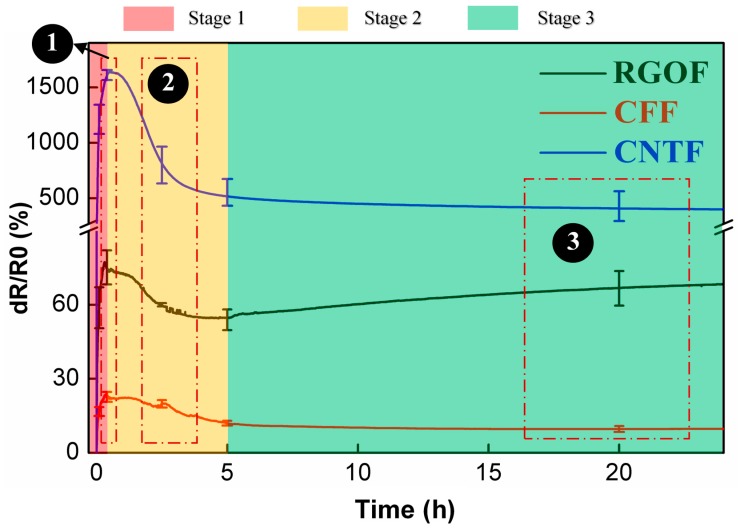
Comparison of the sensing performance of the CNTF, reduced graphene oxide-enabled fabrics (RGOF), and carbon fiber-enabled fabrics (CFF) for the in-line monitoring of FRP manufacturing.

Taking CNTF as an example, we have discussed the typical piezoresistive behavior of the in-line monitoring in the previous section. To further investigate whether other carbon nanomaterials-enabled fabrics also have similar sensing performance and reveal the mechanisms contained, a comparative analysis of the piezoresistivity of CNTF, reduced graphene oxide-enabled fabrics (RGOF), and carbon fiber-enabled fabrics (CFF) was carried out. As shown in Figure 4, the three types of fabrics all exhibited obvious three-stage piezoresistive behavior, and the outline of each signal curve was also roughly similar. Nevertheless, there were still visible dissimilarities at each stage. To specify, in stage 1, the maximum resistance change of the CNTF (~1600%) was much higher than that of the RGOF (~77%) and the CFF (~24%); in stage 2, the degree of resistance attenuation varied substantially and its order from strong to weak is the CNTF (67.7%, *dR/R0*: from ~1600% to ~517%), the CFF (50%, *dR/R0*: from ~24% to ~12%), and the RGOF (28.6%, *dR/R0*: from ~77% to ~55%) by using the decrement rate of *dR/R0* as a measure; and in stage 3, the *dR/R0* value of *the* RGOF showed a tendency to slowly rise (from ~55% to ~69%), while the CNTF and the CFF almost stayed in a stabilized state.

### 3.4. Structure-Dependent Mechanism of In-line Monitoring

As mentioned above, although the three smart fabrics possessed a substantially uniform piezoresistive behavior, there were still discrepancies in certain details that cannot be ignored, because they may play an important role in taking advantage of the various carbon nanomaterials for the purposes of structural health monitoring..To better interpret the discrepancies with valuable insights, a structure-dependent mechanism has been suggested based on the structural properties of the carbon nanomaterials. It is because the conducting network of the fiber sensors has been formed by carbon nanoparticles and its diversified structure may strongly affect the level of the resin infiltration, as well as the deformation during the resin shrinkage.

**Figure 5:**
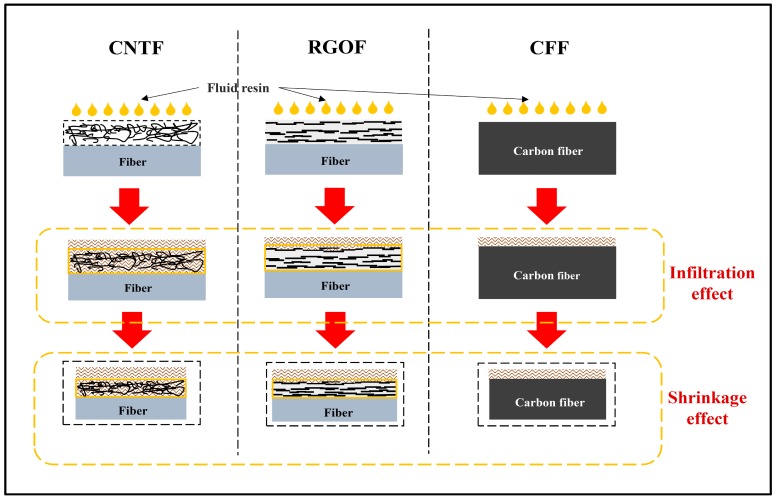
Schematic diagram of the comparison of the CNTF, RGOF, and CFF in the process of composite manufacturing.

To vividly describe our advocated mechanism, a schematic diagram is shown in Figure 5 to demonstrate the complete process from the resin infusion to the full solidification. In stage 1, due to the differences in the microstructures of the various carbon nanomaterial coatings, the level of the resin infiltration to the three types of fiber sensors varies obviously. Because of its fluffy and porous structure, the resin molecules can easily infiltrate and merge into the CNT coating [10]. The adequate flowing space allows them to be in full contact with the CNTs, causing a huge increase in *dR/R0* and improving the upper limit of its growth. Comparatively, the RGO coating is stacked with flake-like graphene particles with large lateral dimensions. The poreless structure leads to a small amount of resin infiltration, and only the surface or upper layers of the conducting network are disturbed by resin. Thus, the improvement of *dR/R0* for the RGOF is weakened. While for the CFF, almost no resin molecules penetrate the highly consistent and densely packed graphite structures with negligible pores. The resin flow just causes a slight volumetric change in the conductive structure and limited interruption of the contact points between the adjacent filaments by squeezing, impacting, and rubbing the carbon fibers. As a result, the sensitivity is reasonably the lowest.

The impact of the varied resin infiltration in stage 1 also extends to the second stage. For the CNTF whose conductive particles are completely impregnated, the volume shrinkage derived from the drastic resin crosslinking will generate strong multidirectional stress to promote tight junctions between the adjacent CNTs. In contrast, there are few resin molecules inside the RGO coating or CFF, such that most conductive particles are not in direct contact with resin matrix. As a result, the shrinkage effect mainly comes from the surface of the structure whose strength, as well as the triggered deformation, is less than that of the CNTF. Thus, the degree of resistance change attenuation of the CNTF is inevitably stronger than that of the other two fabrics. As for why the *dR/R0* value of the CFF has more attenuation than that of the RGOF, we suppose that it may be closely related to the numerous tiny defects on the graphene flakes based on recent representative studies [10,43,45]. Specifically, during the process of the resin shrinkage, a competing behavior in the resistance change of the RGOF is proceeded, due to the combined effect of the compaction resulting from the drastic crosslinking and concentrated internal stress around the defects, which can cause an increase in the tunnel resistance by strengthening the stress intensity and even introducing new defects and voids [23]. Although the piezoresistive behavior indicates that the shrinking effect dominates this competition, defects from the graphene itself have somewhat curbed the momentum of the resistance attenuation. For the CFF, the CF’s consistent and densely packed graphite structure with invisible defects cannot achieve the competing behavior. Its resistance change attenuation is, therefore, more than that of the RGOF.

In stage 3, the shrinkage effect from last stage has almost faded away. For the CNTF and the CFF, because the continuous improvement in the hardness and strength of the upcoming composite cannot further interrupt the configuration of the conducting network, the *dR/R0* value levels off in the whole process. However, for the RGOF, even though the stress concentration effect is overshadowed in the hardening stage, it finally works here. Its unique tiny defects appear to be particularly sensitive to the internal structural stress. By enlarging the stress intensity to disturb the sensing structure, they can help to achieve a continuous rising electrical signal.

Based on all the results and analysis above, we finally conclude that there is a structure-dependent sensing mechanism of the CNTF, RGOF, and CFF in each stage of the composite manufacturing. In the gelation stage, the packing structure of the various fiber sensors dominates the level of the resin infiltration, leading to an increased resistance change with different upper limits. Benefitted by the great advantage of a fluffy and porous conducting network, which can be easily impregnated, the CNTF possesses the highest sensitivity. Because of its impenetrable continuous graphite structure, the CFF exhibits the lowest sensing response. In the hardening stage, the different levels of the resin infiltration result in different intensities of the shrinkage effect. The CNTF, whose sensing element is completely imprisoned by the resin, has more resistance attenuation than the RGOF and CFF. Compared with the CFF, a competitive relationship between the vacuum compaction and stress concentration originated from many tiny defects, making the RGOF’s resistance decay less. In the post-curing stage, the diminished shrinkage effect competes with the disruption of the conducting network, resulting in the continuous rising (RGOF) or depressing (CNTF, CF) of the resistance.

## 4. Conclusions

In summary, the CNTF, RGOF, and CFF were fabricated by respectively weaving CNT- and RGO-coated fibers and carbon fibers into glass fiber fabrics for the in-line monitoring of FRPs. The structural properties of the various fiber sensors were systematically studied through SEM and Raman analysis. With the piezoresistive behavior divided into the gelation stage, hardening stage, and post-curing stage, a detailed comparison of the sensing performance of the CNTF, RGOF, and CFF was performed. Combining the significant piezoresistive dissimilarities found at each stage with the clear differences in the packing structure, we have been more convinced that there is a structure-dependent mechanism dominating the sensing performance of the varied smart fabrics. In the gelation stage, the different levels of the resin infiltration cause the different upside potentials of the resistance changes. In the hardening stage, the resin shrinkage effect results in the resistance attenuation. In the post-curing stage, massive defects on the conducting network can make smart fabrics more sensitive to internal stress, leading to continuous rise of the electrical signal.
